# Transcriptome analysis of peripheral whole blood identifies crucial lncRNAs implicated in childhood asthma

**DOI:** 10.1186/s12920-020-00785-y

**Published:** 2020-09-18

**Authors:** Peiyan Zheng, Chen Huang, Dongliang Leng, Baoqing Sun, Xiaohua Douglas Zhang

**Affiliations:** 1 Department of Allergy and Clinical Immunology, State Key Laboratory of Respiratory Disease, National Clinical Research Center for Respiratory Disease, Guangzhou Institute of Respiratory Health, The First Affiliated Hospital of Guangzhou Medical University, Guangzhou, Guangdong China; 2 CRDA, Faculty of Health Sciences, University of Macau, Taipa, Macau China; 3 Department of Biostatistics, Yale School of Public Health, New Haven, CT 06510 USA

**Keywords:** Childhood asthma, Deep RNA-sequencing, Transcriptome, Long non-coding RNAs

## Abstract

**Background:**

Asthma is a chronic disorder of both adults and children affecting more than 300 million people heath worldwide. Diagnose and treatment for asthma, particularly in childhood asthma have always remained a great challenge because of its complex pathogenesis and multiple triggers, such as allergen, viral infection, tobacco smoke, dust, etc. It is thereby great significant to deeply investigate the transcriptome changes in asthmatic children before and after desensitization treatment, in order that we could identify potential and key mRNAs and lncRNAs which might be considered as useful RNA molecules for observing and supervising desensitization therapy for asthma, which might guide the diagnose and therapy in childhood asthma.

**Methods:**

In the present study, we performed a systematic transcriptome analysis based on the deep RNA sequencing of ten asthmatic children before and after desensitization treatment, including identification of lncRNAs using a stringent filtering pipeline, differential expression analysis and network analysis, etc.

**Results:**

First, a large number of lncRNAs were identified and characterized. Then differential expression analysis revealed 39 mRNAs and 15 lncRNAs significantly differentially expressed which involved in two biological processes and pathways. A co-expressed network analysis figured out a desensitization-treatment-related module which contains 27 mRNAs and 21 lncRNAs using WGCNA R package. Module analysis disclosed 17 genes associated to asthma at distinct level. Subsequent network analysis based on PCC figured out several key lncRNAs probably interacted to those key asthma-related genes, i.e., LINC02145, GUSBP2. Our functional investigation indicated that their functions might involve in immune, inflammatory response and apoptosis process.

**Conclusions:**

Our study successfully discovered many key noncoding RNA molecules related to pathogenesis of asthma and relevant treatment, which may provide some clues for asthmatic diagnose and therapy in future.

## Background

Asthma is a chronic disease of the airways of the lungs, characterized by various symptoms, such as wheezing, coughing, chest tightness, shortness of breath, dyspnea, etc. [1, 2]. It has become one of the most common long-term conditions in children and severely affects the quality of life of children as well as their parents. In Europe, prevalence rates of asthma among children increases from 5% in Albania to 21% in the UK. 9.1% of US children (6,7 million) had asthma in 2007 [1]. In China, the prevalence of childhood asthma increased extremely since 1990s, which ranged from 0.93% in 1990 to 1.54% in 2000 [3]. There is no definitive explanation for this increase, even though the prevalence of asthma in children has increased over decades. More importantly, there is no full cure is available up to now despite majority of children with asthma can alleviate the asthmatic symptoms and obtain adequate asthma control via avoidance of triggering factors, rational management and/or medication, such as short-acting inhaled β2-receptor agonists [4, 5]. However, a small proportion (~ 5%) of asthmatic children have uncontrolled asthma despite maximum medical treatment [6]. In addition, diagnosing asthma in children faces challenge as well, e.g., a number of childhood conditions exhibit compatible symptoms to those caused by asthma, such as shortness of breath, wheezing and cough [5]. To further complicate the issue, those conditions can coexist with asthma and confuse the evaluation of patients.

Like other immune-related diseases, genetic components, i.e., sequences variants, in combination with environmental factors, i.e., allergen, viral infection, may contribute to the development of asthma [7–9]. Concretely, genome-wide association studies have detected numerous asthma-associated gene variants, while few of them (less than 10%) can be explained to contribute to the risk of asthma [10]. On the other hand, environmental factors have been reported to modulate epigenetic modifications and thereby affect gene expression and phenotype [11, 12]. Epigenetic mechanisms have been reported to be key to functions of airway smooth muscle (ASM), e.g., aberrant phenotype of ASM lead to the obstruction and inflammation of airway [13]. Therefore, diverse cellular compartments orchestrated by multiple environment-driving factors make the mechanisms underlying asthma extraordinary complex and unclear, particularly with respect to long noncoding RNAs (lncRNAs).

lncRNAs have been reported to possess multiple functions including regulation of gene expression, transcriptional activation and silencing of genes and thus play critical roles in a diversity of cellular process [14, 15]. Aberrant expression of many specific lncRNAs was reported to be correlated to the pathogenesis of various diseases in human and model animals [16, 17]. Furthermore, the roles of lncRNAs implicated in asthma have been emerged recently. Several studies achieved by whole genome RNA-seq have identified many lncRNAs specifically expressed in T cells at varying stages of development and differentiation [18]. For instances, the lncRNA named Tmevpg1 was found to be uniquely expressed by the T helper cell 1 (TH1) and is essential to the transcription of Ifng by TH1 subset [19]. The lncRNA GATA3-AS1 transcribed in the opposite direction from GATA3 was reported to be co-expressed with GATA3 in mouse and human TH2 cells [20]. TH2 cells responses have already demonstrated to link to both allergy and asthma. However, the gaps between the function of the lncRNAs specifically expressed in immune system or T lymphocytes and asthma still unfilled and require further deep investigation, e.g., despite GATA3-AS1 may play a role in asthmatic response, its functions are unknown. Here, to attempt to fill some of those gaps and identify the novel lncRNAs which might be useful molecules for observing and supervising desensitization therapy for childhood asthma, we performed a systematic transcriptome analysis based on the deep RNA-seq data of peripheral whole blood of ten asthmatic children before and after desensitization treatment.

## Results

### Identification and characteristics of lncRNAs

At first, the raw data was filtered and trimmed to eliminate the adaptor sequences and low-quality reads using Trimmomatic. This process yielded an average of 121,690,584 clean reads for each sample (Table S1). Then the clean reads were utilized to re-construct the new transcriptome for human for the present study. Firstly, all the clean data were aligned to human genome sequences using STAR (Table S2). After that, the aligned bam files were subjected to StringTie to assemble into 215,331transcripts (Table S3). We simply compared these assembled transcripts against the known transcripts of human derived from Ensembl database via BLASTn, the result showed that 16,220 (~ 7.5%) novel transcripts were identified in the present study.

**Fig. 1:**
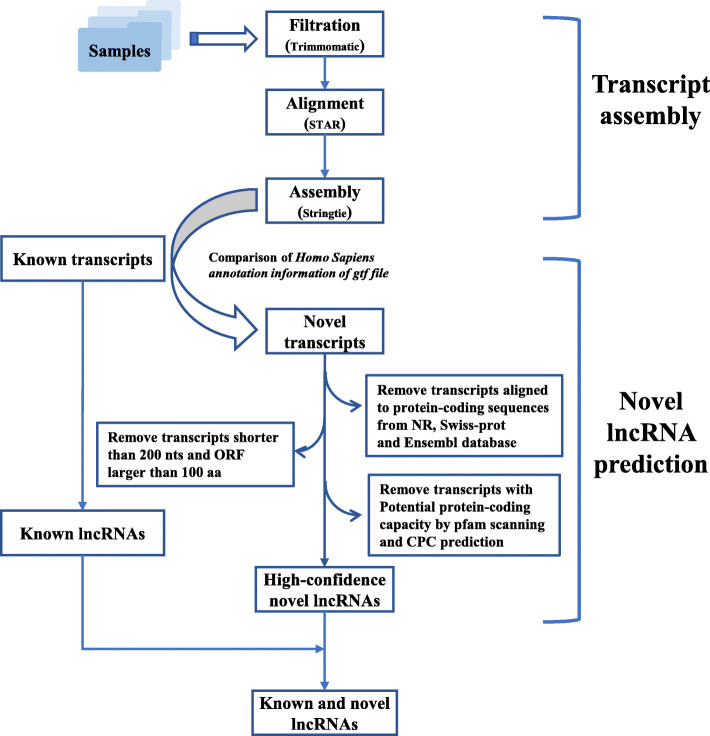
The bioinformatics pipeline used for the identification of lncRNAs for ten asthmatic children

**Fig. 2:**
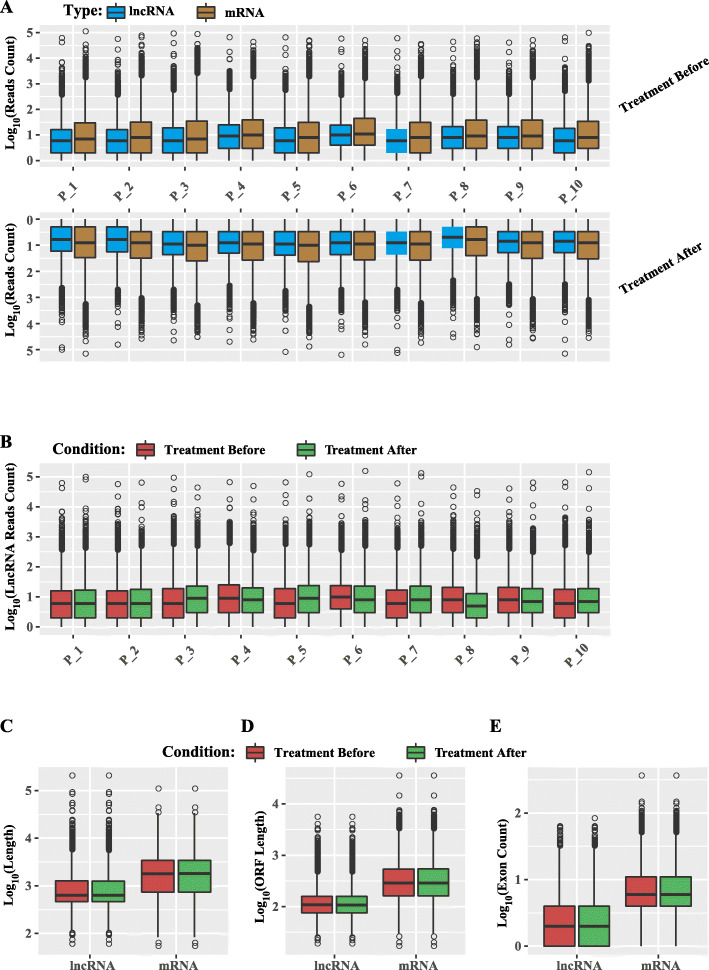
General characteristics of assembled transcripts in ten asthmatic children between two different conditions. **a** Expression level indicated by log_10_ (reads count) in mRNAs and lncRNAs in for ten asthmatic children before and after treatment. **b** Expression level indicated by log_10_ (reads count) in lncRNAs between two different conditions. **c** Distribution of transcript length by log_10_ (Length) **d** ORF length by log_10_ (ORF Length) **e** Exon count by log_10_ (Exon Count) in mRNAs and lncRNAs in in ten asthmatic children

To identify high-confidence lncRNAs for this study, we initially extracted the known lncRNAs based on the annotation information of gtf file of *Homo sapiens* (GRCh38.p10) from Ensembl database (details see Methods). As a result, a total of 52,791 known lncRNAs were detected. On the other hand, we applied a stringent stepwise filtering pipeline (Fig. 1) to identify novel lncRNAs. This analysis generated 5310 novel lncRNAs (Table S4). Furthermore, aimed at assessing the quality of identified lncRNAs, we performed a comparative study between lncRNAs and mRNAs before and after treatment in the transcript length, exon counts, open reading frame (ORF) length and expression level (Fig. 2). The results indicated that there was no obvious difference of expression level among these patients no matter in mRNAs or lncRNAs (Fig. 2a). The similar situation exists in the comparison of expression before and after treatment (Fig. 2b). In consistent patterns of with previous studies of eukaryotic lncRNAs, the length of the majority of identified lncRNAs and the corresponding ORF was much shorter than that of mRNAs (Fig. 2c, d), and the exon count of the lncRNAs was also less than that of mRNAs (Fig. 2e).

**Fig. 3:**
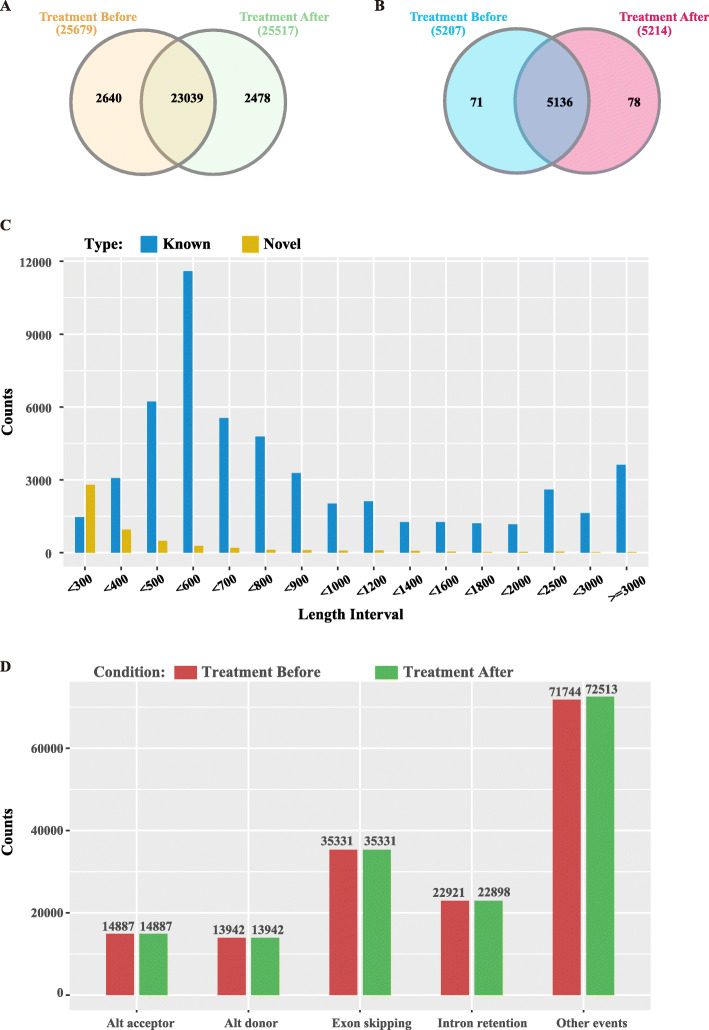
General features of lncRNAs in ten asthmatic children before and after treatment. **a** Comparison of known lncRNAs before and after treatment. **b** Comparison of novel lncRNAs before and after treatment. **c** Distribution of length of known and novel lncRNAs. **d** Distribution of five different types of alternative splicing events in two different status

Moreover, we found that a small proportion of known and novel lncRNAs specifically expressed in distinct status (Fig. 3a, b). In addition, the length of majority of known lncRNAs ranges from 500 bp to 700 bp, whereas the length of lncRNAs were found mainly shorter than 300 bp (Fig. 3c). We also detected five main alternative splicing (AS) modes for these transcripts using AStalavista [21]. The results showed that there was no significant discrepancy in those five AS modes before and after treatment (Fig. 3d).

### Differential expression analysis reveals desensitization-treatment-related module

**Fig. 4:**
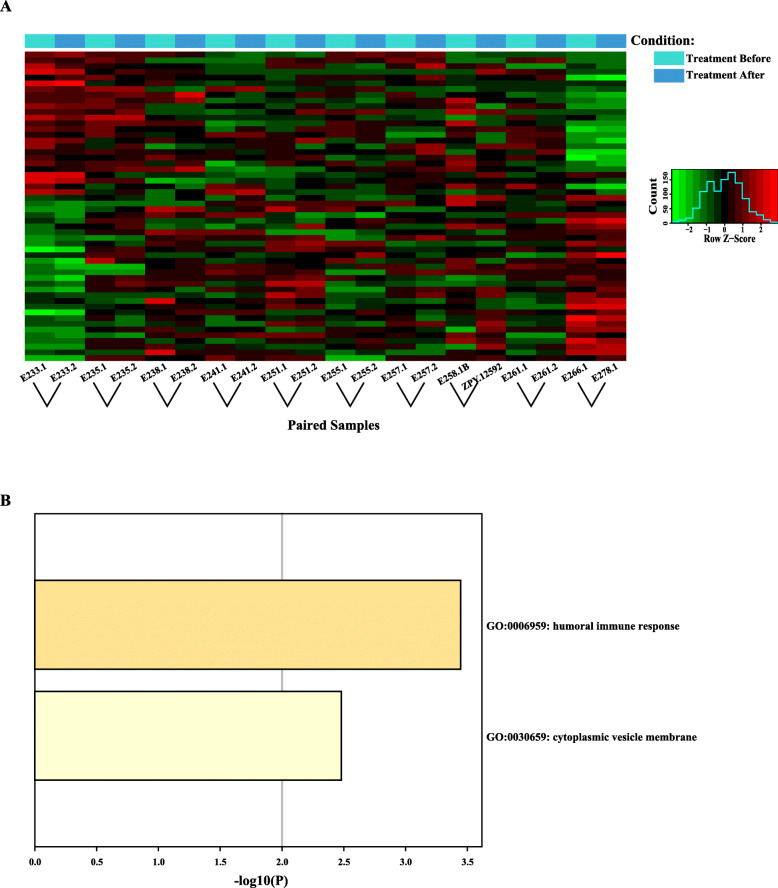
Differential expression analysis between before treatment and after treatment. **a** Heatmap of expression profiles using differentially expressed transcripts. The heatmap was visualized using R package gplots, all expression values were normalized using Z-score using R package DESeq2. **b** Functional GO enrichment analysis based on differentially expressed transcripts using Metascape

To investigate the lncRNAs as well as their possible roles implicated in asthma before and after treatment, we initially performed paired differential-expression analysis using DESeq2 package for R [22]. The results indicated that only 54 transcripts (15 lncRNAs and 39 mRNAs) were significantly differentially expressed in the patients after treatment (Table 1, Fig. 4a). After that, all the differentially expressed transcripts were subjected to metascape to search for possible functional biological processes. As a result, two biological processes were significantly enriched, including humoral immune response and cytoplasmic vesicle membrane (Fig. 4b).

**Table 1:** Differentially expressed lncRNA identified by comparison ten asthmatic children before treatment and after treatment

Id	baseMean	log2FC	lfcSE	stat	pvalue	padj
ENST00000361696	11.94749	−3.68586	0.849001	−4.34141	1.42E-05	0.019371
MSTRG.201.1	11.18279	−3.27319	0.803233	−4.07502	4.60E-05	0.043952
MSTRG.576.10	3.482717	−4.16184	0.906494	−4.59114	4.41E-06	0.008807
ENST00000444082	27.83899	3.194938	0.770347	4.147399	3.36E-05	0.03728
ENST00000455621	36.36187	−3.76744	0.70904	−5.31343	1.08E-07	0.000419
MSTRG.3834.1	115.1273	2.675254	0.553098	4.836857	1.32E-06	0.002889
MSTRG.3835.1	144.4785	2.334084	0.479032	4.872501	1.10E-06	0.002789
MSTRG.3949.7	94.58607	4.078681	0.894632	4.559059	5.14E-06	0.009291
ENST00000469464	22.2803	4.246452	0.875138	4.852323	1.22E-06	0.002808
ENST00000619625	67.74338	−6.6478	0.748755	−8.87848	6.78E-19	3.43E-14
ENST00000542875	22.3978	−3.33454	0.739618	−4.50847	6.53E-06	0.010664
ENST00000390543	490.4011	−3.13372	0.74515	−4.20549	2.61E-05	0.03217
ENST00000632950	26.95596	−4.48368	0.928419	−4.82937	1.37E-06	0.002889
MSTRG.12440.9	588.6879	3.849564	0.877689	4.386025	1.15E-05	0.017154
MSTRG.12440.14	239.4075	4.319831	0.869486	4.968257	6.76E-07	0.001913
MSTRG.14077.1	12.51064	3.46116	0.846337	4.089576	4.32E-05	0.042076
ENST00000392730	648.8246	−2.08722	0.500413	−4.17099	3.03E-05	0.035709
ENST00000617914	21.30579	−2.55837	0.630262	−4.05922	4.92E-05	0.046163
ENST00000262407	232.6204	2.179113	0.497495	4.380172	1.19E-05	0.017154
MSTRG.16347.5	67.00047	3.220476	0.581223	5.540857	3.01E-08	0.000139
MSTRG.16364.2	364.4441	2.804439	0.476558	5.884778	3.99E-09	2.24E-05
MSTRG.16381.10	16.27998	−3.55432	0.85817	−4.14174	3.45E-05	0.03728
MSTRG.19617.1	275.7269	−2.61749	0.634407	−4.12588	3.69E-05	0.038159
MSTRG.21274.2	456.3239	2.216572	0.53804	4.119715	3.79E-05	0.038411
ENST00000468494	164.8995	−2.25483	0.499401	−4.51508	6.33E-06	0.010664
ENST00000327097	147.3837	3.161638	0.724294	4.365133	1.27E-05	0.017867
MSTRG.23514.1	15.36723	3.753959	0.865911	4.335271	1.46E-05	0.019396
ENST00000426585	33.62136	3.929173	0.687084	5.718621	1.07E-08	5.44E-05
ENST00000609510	29.43023	3.580698	0.726702	4.927324	8.34E-07	0.002221
MSTRG.24857.1	98.71423	5.023013	0.691356	7.265446	3.72E-13	6.27E-09
MSTRG.26869.6	21.70362	−3.73658	0.815436	−4.58231	4.60E-06	0.008807
ENST00000226524	311.4886	2.578902	0.628229	4.105037	4.04E-05	0.04013
ENST00000507209	159.259	2.189576	0.530265	4.129215	3.64E-05	0.038159
ENST00000515156	81.91006	−3.02593	0.609214	−4.96694	6.80E-07	0.001913
MSTRG.29998.1	62.51979	−4.29729	0.68561	−6.26784	3.66E-10	2.65E-06
ENST00000418981	141.0499	−5.10813	0.634533	−8.05022	8.26E-16	2.09E-11
ENST00000399084	11.48127	4.223662	0.803087	5.259284	1.45E-07	0.000488
ENST00000487676	747.0078	2.989293	0.449852	6.645054	3.03E-11	3.07E-07
MSTRG.32871.1	94.01289	−2.72519	0.656786	−4.14928	3.34E-05	0.03728
ENST00000363062	73.87184	−3.53383	0.669256	−5.28024	1.29E-07	0.000467
MSTRG.33216.1	49.75533	−3.70187	0.741945	−4.98941	6.06E-07	0.001913
ENST00000450062	91.39431	4.140332	0.681522	6.075124	1.24E-09	7.84E-06
ENST00000440744	43.10726	3.437249	0.625981	5.490982	4.00E-08	0.000169
ENST00000327857	427.9353	−4.8754	0.73134	−6.6664	2.62E-11	3.07E-07
MSTRG.37554.1	36.55034	2.568157	0.56099	4.577902	4.70E-06	0.008807
MSTRG.37556.1	37.44479	3.047537	0.692021	4.403824	1.06E-05	0.016827
ENST00000432132	238.6451	2.999853	0.456699	6.568562	5.08E-11	4.29E-07
MSTRG.38128.1	33.87995	−2.93775	0.691359	−4.24924	2.15E-05	0.027149
ENST00000432272	18.51986	−3.45454	0.811905	−4.25486	2.09E-05	0.027149
MSTRG.38731.1	92.82106	−2.67844	0.639978	−4.18521	2.85E-05	0.034344
ENST00000381106	24.44047	−3.29982	0.796903	−4.14081	3.46E-05	0.03728
ENST00000434837	17.31914	4.057187	0.835584	4.855509	1.20E-06	0.002808
ENST00000404689	5.124954	3.904176	0.889565	4.38886	1.14E-05	0.017154
ENST00000331428	9.976701	4.000181	0.881455	4.538155	5.67E-06	0.009907

### Co-expression network analysis reveals desensitization-treatment-related module

**Fig. 5:**
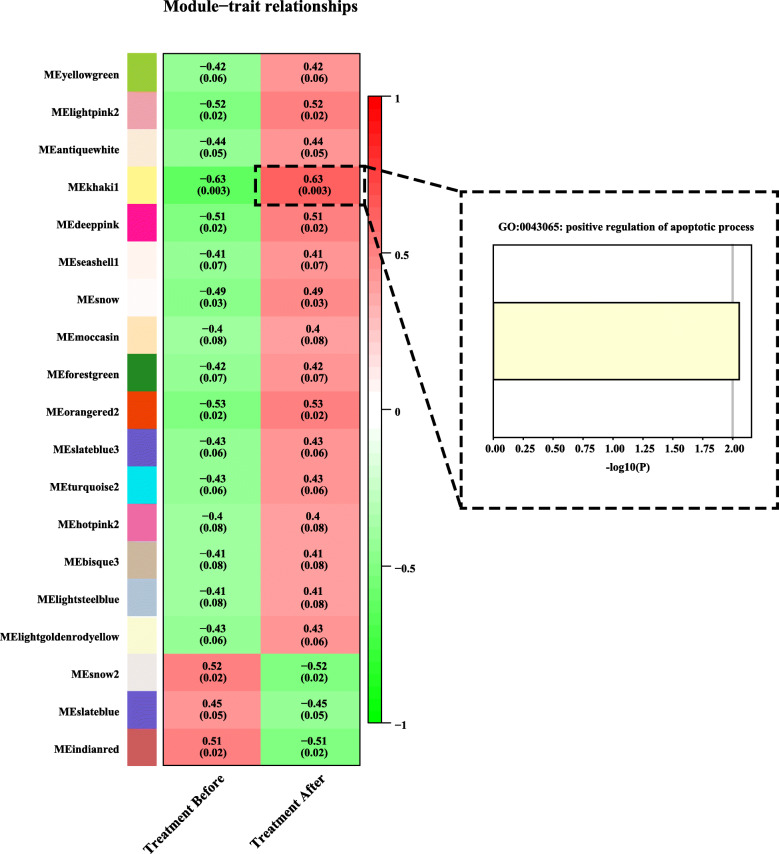
Detection of treatment-related module. **a** Module-trait associations. Each row corresponds to a module eigengene, column to a trait. Each cell displays the corresponding correlation and *p*-value. Dotted frame represents the biological process significantly enriched by the transcripts within the treatment-related module via Metascape

Alternatively, we utilized a more sensitive and robust approach to explore the lncRNAs implicated in asthma, i.e., co-expression network analysis, which was achieved via WGCNA in R. Firstly, the expression profile of all transcripts was assessed using featurecounts and normalized by DESeq2 package for R. Then the cluster analysis was conducted using flash tools package of WGCNA to detect outliers of samples. The results indicated that all samples were well clustered and passed the cuts (Figure S1). A network-topology analysis based on different soft-thresholding powers was performed to get relative balance scale independence and mean connectivity of WGCNA. The optimal power for the scale-free topology fit index was calculated out for construction of hierarchical clustering tree (Figure S2). This analysis clustered all the correlated transcripts into 80 modules with strong correlation with trait (Figure S3). Each module contains independent datasets of transcripts (includes mRNAs and lncRNAs). The interactions among those modules were visualized in Figure S4 with randomly selected transcripts. After that, the modules significantly associated with specific trait (desensitization treatment) was detected using the function *plotEigengeneNetworks* of WGCNA (Table 2). The results showed that a module (khaki1) were significantly correlated to the trait (desensitization treatment) (Fig. 5), which contains 27 mRNAs and 21 lncRNAs. The relationship of transcript significance with module membership was visualized in Figure S5.

**Table 2:** Modules identified with high correlation by WGCNA

Module	Number of transcripts
yellowgreen	495
slightsteelblue	237
deeppink	147
lightpink2	138
slateblue	129
moccasin	107
antiquewhite	84
indianred	80
lightgoldenrodyellow	75
slateblue3	72
bisque3	70
turquoise2	69
orangered2	54
snow	53
hotpink2	52
khaki1	48
seashell1	47
snow2	47
forestgreen	44

### Functional analysis of module reveals key genes related to asthma

In order to investigate the potential relationship of transcripts which co-localizes in desensitization-treatment-related module with asthma treatment, the annotated transcripts (mRNAs) of the module were subjected to a gene annotation server Metascape for functional enrichment analysis. The result showed that one biological process (GO:0043065: positive regulation of apoptotic process) was significantly enriched (Fig. 5) by four genes: TRIO, PEA15, KLRK1, NDUFA13.

In addition to these four apoptosis-related genes (TRIO, PEA15, KLRK1, NDUFA13), we also found that the other genes in desensitization-treatment-related module exhibiting distinct degree of correlations to asthma via literature review [23–37]. Concretely, four genes (ATF7IP, SLC43A3, AKAP7, HMGN1) were found to be correlated to pathogenesis or treatment of asthma in immune. For instance, ATF7IP can form a protein-complex by binding to MBD1 (methyl CpG binding protein 1) and Aire (autoimmune regulator) to maintain immune tolerance [23]. Immunological tolerance to self-antigens is critical to the prevention of autoimmune disease, including asthma. In addition, two genes (ELMO2, WDFY1) were demonstrated to play roles in inflammation response of asthma. ELMO2 was found as an ILK-binding protein to play key role in integration of adhesion and migration pathways. Abnormal regulation of migration is associated to chronic autoinflammatory disorder, such as asthma [33]. Furthermore, two genes (G2E3, ZNF19) were found to be differentially methylated between asthmatics and non-asthmatics [34, 35]. Combined with four apoptosis-related genes, all those asthma-related genes could be summarized in Table 3.

**Table 3:** Summary of 17 key asthma-related genes located in desensitization-treatment-related module

Category	Gene	Description of correlation with asthma	References
Immune correlation	ATF7IP	ATF7IP can interact with methyl CpG binding protein 1 (MBD1) and the autoimmune regulator (Aire) protein to maintain immune tolerance	Michael Waterfield et al. [31]
	SLC43A3	SLC43A3 was found to be associated with IgE level by epigenome-wide approach, meta-analysis	Youming Zhang [32]; Liang et al. [33]
	AKAP7	AKAP7 encodes protein family binding to a regulatory subunit (RII) of cAMP-dependent protein kinase A (PKA); sElevation of cAMP leads to the activation of both PKA and Epac and thereby modulates airway smooth muscle responses. It has been reported that coupling of the β2-adrenoceptor to Gi leads to activation of ERK signaling.	W J Poppinga et al. [34]
	HMGN1	The nucleosome-binding protein HMGN1 is a potent alarmin that binds TLR4 and induces antigen-specific Th1 immune responses.	Feng Wei et al. [35]
Inflammation response	ELMO2	ELMO2 can interact ILK to play key role in the integration of adhesion and migration pathways, migration is closely association to autoinflammatory disorder, i.e., asthma	Ernest Ho and Lina Dagnino [38]
	WDFY1	WDFY1 mediates TLR3/4 signaling by recruiting TRIF. Positively regulates TLR3- and TLR4-mediated signaling pathways by bridging the interaction between TLR3 or TLR4 and TICAM1. Promotes TLR3/4 ligand-induced activation of transcription factors IRF3 and NF-kappa-B, as well as the production of IFN-beta and inflammatory cytokines	Andriana I. Papaioannou et al. [36]; Q. Zhang et al. [37]
Methylation	G2E3	G2E3 was found in top-ranked DMPs (differentially methylated positions) in asthma-discordant MZ twins	Therese M. Murphy et al. [39]
	ZNF19	ZNF19 was found in Top fifty promoter loci with differential methylation between obese asthmatics and obese non-asthmatics	Deepa Rastogi et al. [40]
Apoptosis	NDUFA13	Differential expression analysis indicated NDUFA13 was differentially expressed between healthy controls and allergic rhinitis patients.	Wagener, A.H. et al. [23]
	KLRK1	NKG2D is encoded by KLRK1 gene. NK cells promote allergic pulmonary inflammation in response to HDM allergen by mechanisms dependent on NK-cell intrinsic expression of the major activating receptor NKG2D. Klrk1^−/−^ mice exhibited a profoundly impaired inflammatory response to allergen challenge compared with klrk1^+/+^ mice.	Nazanin Farhadi et al. [41]
	PEA15	PEA15 is an important protein that regulates death receptor induced apoptosis and proliferation signaling by binding to FADD and relocating ERK1/2 to the cytosol. ERK1 is important for Th2 differentiation and development of experimental asthma.	Stéphane Kerbrat et al. [29]; Nicholas Goplen et al. [42]
	TRIO	significantly enriched in the process of positive regulation of apoptosis	–
Others	WWP2	detected to be differentially expressed from HBEC (human bronchial epithelial cells) ALI cultures stimulated with IL-6/sIL-6R compared to non-stimulated control.	Zala Jevnikar et al. [43]
	VPS37A		
	GPR160		
	VWA5A		
	SPDYE6		

### Network analysis based on PCC reveals key lncRNAs implicated in asthma

**Fig. 6:**
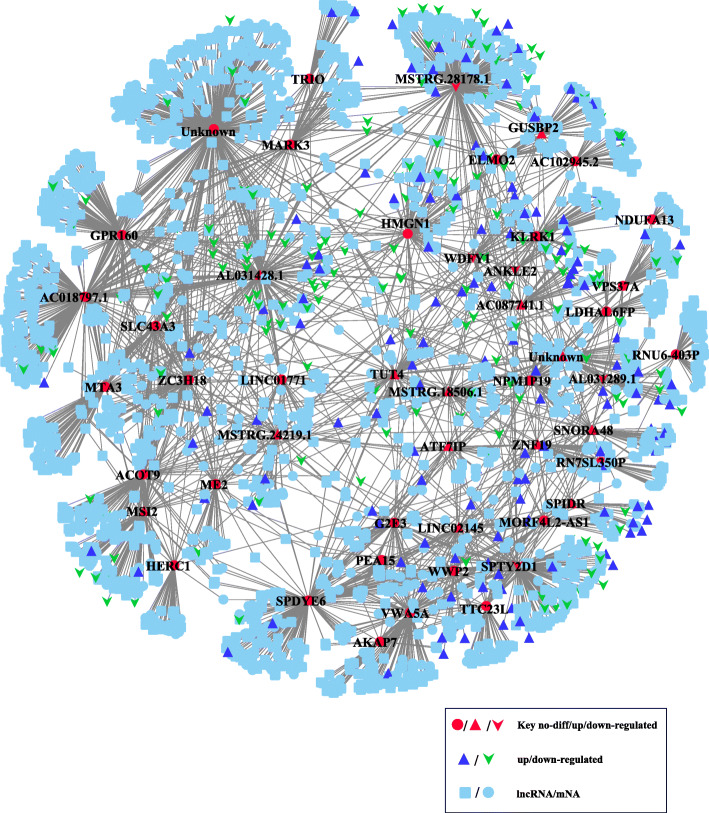
Network analysis defined asthma-related interactome in desensitization treatment-related module. The mRNA-lncRNA interactions was defined using PCC (Pearson Correlation Coefficient) methods and visualized using Cytoscape

Our aforementioned analysis has successfully assisted us to identify several asthma-related genes in desensitization-treatment-related module. However, the correlation of lncRNAs with asthma in the module were still unknown. For that, we performed a network analysis to establish RNA-RNA interaction network for all the 48 transcripts (including mRNAs and lncRNAs) in desensitization-treatment-related module based on the remaining 102,041 assembled transcripts using pearson correlation coefficient (PCC) method. The RNA-RNA pairs which had absolute value of pearson correlation ≥0.7 and *P*-value< 0.05 were retained. This analysis detected a total of 3502 RNA-RNA interaction relationships including mRNA-mRNA, mRNA-lncRNA, which involved 1654 mRNAs and 864 lncRNAs (Fig. 6).

**Fig. 7:**
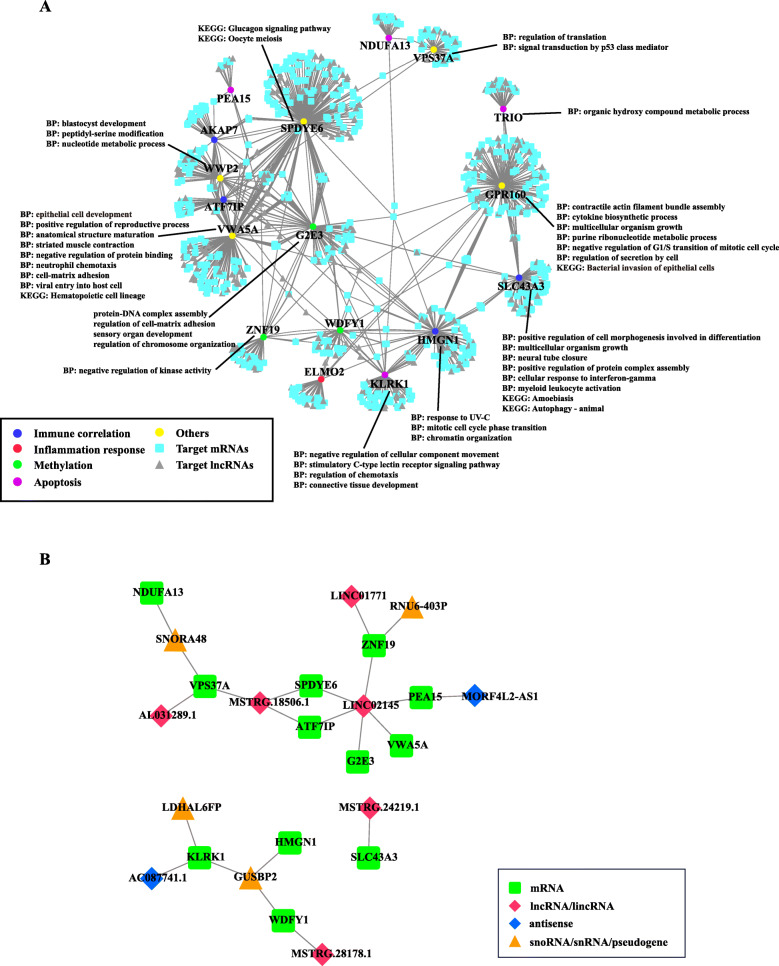
Sub-network indicating key lncRNA related to asthma. **a** Sub-network of mRNA-lncRNA interactions involved in 17 key asthma-related genes. **b** Core sub-network displaying interactions in which both sides located in desensitization treatment-related module

We further concerned which lncRNAs target to the 17 asthma-related genes as well as their possible roles in asthma. Since that, we extracted a sub-network of these 17 asthma-related genes from the above network (Fig. 7a). This step indicated that 323 lncRNAs exhibited potential correlations to these asthma-related genes, implying they may play roles in pathogenesis and treatment of asthma. To further investigate their possible functions implicated in asthma, the target mRNAs for each asthma-related gene were extracted to be subjected to Metascape server for functional analysis. The results showed that majority of target mRNAs of asthma-related gene could significantly enriched by diverse biological processes (Fig. 7a).

Among them, many RNA-RNA interactions aroused our attentions since both sides of interaction co-localizes in the desensitization-treatment-related module (Fig. 7b), which suggested their strong correlation with asthma. Concretely, three known lncRNAs (LINC01771, LINC02145, AL031289.1) and three novel lncRNAs (MSTRG.24219.1, MSTRG.28178.1, MSTRG.18506.1) were found to interact with many asthma-related genes. Notably, LINC02145 exhibited to be correlated to six asthma-related genes, including ZFN19, G2E3, ATF7IP, PEA15, SPDYE6, VWA5A. Despites there is no clinical significance was reported in this known lncRNA, our analysis indicated that its strong association with asthma before and after desensitization treatment. A similar situation was found in a novel lncRNA MSTRG.18506.1 which interacts with three asthma-related genes, i.e., SPDYE6, ATF7IP, VPS37A. Interestingly, in addition to lncRNAs, we found some other types of noncoding RNAs exhibited correlations with asthma-related genes, including antisense, snoRNA, snRNA and pseudogene. To test our findings that those specific lncRNAs and mRNAs probably associated to immune/inflammatory-related genes in asthma, we selected three lncRNA/mRNAs, including KLRK1, LINC02145, GUSBP2, which exhibits differential expression before and after treatment for the qRT-PCR. The results were demonstrated to be consistent to our bioinformatic analysis (Figure S7).

## Discussion

With the advancement of high throughput sequencing technology, tens of thousands of lncRNAs have been detected in human. Despite the function of majority of lncRNAs are unknown so far, their important roles in diverse biological processes has already been demonstrated, particularly close correlations to diseases. In the present study, we detected at least one order of magnitude lncRNAs larger than those identified in our previous studies about lncRNAs [38, 42, 43]. One of reason is the strategy of RNA sequencing in our previous studies is to capture the RNAs with polyA tail (mRNAs), which could only identify around half of lncRNAs. Here we applied total RNA sequencing technology to capture all linear RNAs, which contains most of lncRNAs. In addition, we improved the analysis pipeline for identification of lncRNAs used in our previous studies, which mainly focused on the identification of novel lncRNAs. Then the differential expression analysis showed that only a small proportion of RNAs were differentially expressed in asthmatic patients before and after treatment. The possible reason is that asthma as a chronic respiratory disease, treatment of it might not directly be reflected in peripheral blood. The RNA change triggered by treatment in asthmatic patients might mainly occur in airway epithelium. This is one limitation of our study. This limitation could be solved by using the deep RNA sequencing data of airway epithelium cell. Another limitation to be noted is that ten individuals participated in the present study are composed of nine male children and one female child. To our knowledge, gender, in particular during pubertal period (one female child in this study is 15 years old) might affect the results of differential expression analysis. To be clear, the results and the conclusions in the present study are based mainly on male population of asthmatic childhood patients.

Regardless, the functional enrichment analysis of differentially expressed transcripts indicated their functions might be involved in humoral immune response. Humoral immune response is one of two main arms of the immune system, which triggers specific B cells to proliferate and secrete large amounts of their specific antibodies. This process attracts a helper T (T_H_) cell to be activated against a particular antigen, which implying its importance in immune response to asthma.

After that, co-expression network analysis was achieved by WGCNA package to identify desensitization-treatment-related module. Although correlation value of the most trait-related (khaki1) module is not very significant (only reach 0.63) compared to other WGCNA studies, this analysis successfully figured out several mRNAs and lncRNAs correlated to asthma treatment. Functional analysis on these transcripts within the desensitization-treatment-related module revealed four genes (TRIO, PEA15, KLRK1, NDUFA13) significantly enriched in the positive regulation of apoptotic process. This finding actually was consistent to previous studies for asthma [39–41]. Concretely, KLRK1 encodes NKG2D, and aberrant expression of NKG2D ligands has been reported in sites of inflammation and in tissues undergoing autoimmune pathologies, including asthma [44]. Also, Klrk1^−/−^ mice exhibited a profoundly impaired inflammatory response to allergen challenge compared with klrk1^+/+^ mice [36] . The qRT-PCR experiment for KLRK1 based on our samples indicated that the expression of KLRK1 was up-regulated after desensitization treatment, which might alleviate the inflammatory response and respiratory symptoms. This result was consistent to our differential expression analysis, despites the expression difference is not very statistically significant (*p* value = 0.09676, Figure S7-B). The possible reason may be that we just used three individuals for the qRT-PCR experiments, the sample size might limit the statistical power. The reason why we just used three individuals is that the blood sample was preserved too long despites we kept them refrigerated (− 80 °C), the RNAs of some samples are degraded at different degrees. We just found the RNAs of three samples were qualified for qRT-PCR experiments after the quality inspection. Another two transcripts (LINC02145, GUSBP2) for qRT-PCR validation had the same issue. Thereby, it is reasonable that these validations might be more significant if we use ten sample for qRT-PCR experiments. PEA15 has been proved as an important protein that regulates death receptor induced apoptosis and proliferation signaling by binding to FADD and relocating ERK1/2 to the cytosol. It regulates subcellular compartmentalization and activity of phosphoERK, and ERK1 was found to be important for Th2 differentiation and development of experimental asthma [37]. In addition, Sandra Pastorino et al’s study showed that ablation of PEA-15 led to accumulation of ERK in the nucleus in PEA-15/ T cells, which would enhance the T-cell proliferation [45]. In asthma, stimuli can promote repair mechanisms to induce cellular proliferation and inhibits apoptosis, and delayed survival of inflammatory cells may in turn aggravate the respiratory symptoms. In the present study, our analysis indicated that the apoptotic process of asthmatic children was upregulated after desensitization treatment, which implies the asthmatic symptoms in those patients might be improved in different degrees. Notably, our analysis figured out a key lncRNA LINC02145 directly targeted on PEA15 (Fig. 7b). Subsequent qRT-PCR on LINC02145 showed the expression of this lncRNA exhibited significant up-regulation (Figure S7-C) after desensitization treatment, which might affect the expression of PEA15 and further regulates death receptor induced apoptosis and proliferation signaling and relieve the respiratory symptoms.

Additionally, our analysis indicated that a pseudogene GUSBP2 (also defined as lncRNA in GeneCards Suite [46]) was found to interact with KLRK1, as well as another two immune/inflammatory-related genes, including HMGN1 and WDFY1. HMGN1 encodes the nucleosome-binding protein, which was reported as a potent alarmin that binds TLR4 and induces antigen-specific Th1 immune responses [27]. WDFY1 could mediate Toll-like receptor3/4 (TLR3/4) signaling by recruiting TRIF. TLRs were found to be expressed in the lung tissue and some cells of innate and adaptive immune system. Abnormal expression of TLRs could lead to aberrant expression of many inflammatory and anti-inflammatory mediators [36]. These 3 genes thereby have been well demonstrated to be closely associated to immune and inflammatory response in asthmatic status. Regarding GUSBP2, the associations with asthma actually has been reported in a microarray study for asthma, which showed that GUSBP2 was differentially expressed in comparison of asthmatic patients with health controls [47]. However, its detailed mechanisms in asthma was not illuminated. In the present study, our network analysis disclosed GUSBP2 exhibiting correlations to these immune/inflammatory-related genes, implying it might target to these genes to participate the regulation of immune and inflammatory process in asthma. Indeed, GUSBP2 was found as the highest up-regulated fold change of lncRNA in CD (Crohn’s disease) patient plasma via microarray screening [48]. CD is known to us as one of inflammatory bowel diseases, which suggests the existence of strong association between GUSBP2 and inflammatory reaction. In the present study, our qRT-PCR experiment on GUSBP2 indicated that the expression of GUSBP2 was up-regulated after desensitization treatment (Figure S7-A). This finding was also consistent with our differential expression analysis. The result indicated that the expression of GUSBP2 was repressed in asthmatic patients compared to healthy controls (Figure S8). Subsequent network analysis based on PCC method displayed that GUSBP2 might target on KLRK1, whose expression was also already found to be up-regulated after desensitization treatment. Notably, the cutoff of correlation value was not set to traditional threshold (0.9 ~ 0.95) since the correlation between those transcripts in this study is not as strong as our previous lncRNA studies [38, 43]. One of possible reason is that the desensitization treatment might not make a big change of RNAs in peripheral blood of these asthmatics, which further led to weak correlations among those RNAs. This also is the reason that it is not easily to figure out key RNAs related to asthma or treatment simply via differential expression analysis, thereby we applied co-expression network analysis, a relatively robust and sensitive strategy for our study. Our findings indicated that the desensitization treatment might induce GUSBP2 positively work with KLRK1 to alleviate the inflammatory response and the respiratory symptoms. To validate this further, we analyzed a expression data which derives from GEO database (accession number: GSE2125), which sequenced 45 alveolar macrophages from human subjects (30 non-smoker healthy people and 15 asthmatic patients). The result showed that both genes exhibited a higher expression level in healthy controls (Figure S8). Subsequent study on relationship between GUSBP2 and KLRK1 using PCC method indicated that they have a moderate correlation (PCC value = 0.538), which was consistent with It was also implied that GUSBP2 and KLRK1 might play important roles in the occurrence of asthma and might be potential useful RNA molecules for therapy and diagnosis in asthma.

## Conclusions

Our network analysis based on the deep RNA sequencing of ten childhood asthmatics before and after desensitization treatment disclosed several lncRNAs exhibiting close correlation to the asthma-related genes, particularly involved in immune, inflammatory response and apoptosis process. This finding provides many promising key noncoding RNA, i.e., LINC01771, LINC02145, GUSBP2, etc. which might be served as novel molecules for observing and supervising desensitization therapy for childhood asthma.

## Methods

### Sampling and deep sequencing

All individuals consented, and the project were approved by the institutional review boards of the Guangzhou Medical University, University of Macau. Ten children diagnosed with asthma in the 1st Affiliated Hospital of Guangzhou Medical University, China were selected for our study. Of the allergic asthma patients had received *Dermatophagoides pteronyssinus* (Der p) SCIT (before treatment and after half a year). All patients completed questionnaires on their demographics and medical history and fulfilled the Allergic Rhinitis and its Impact on Asthma (ARIA) criteria for allergic rhinitis and/or the Global Initiative for Asthma (GINA) criteria [49] for mild-to-moderate asthma, sIgE to Der p, and *Dermatophagoides farinae* (Der f). The detailed information for these patients was summarized in Table 4. For each individual, peripheral whole blood was extracted and then the peripheral blood mononuclear cell (PBMC) was separated from it using ficoll-paque. After that, the total RNA was extracted using trizol (invitrogen) method and the RNA quality was assessed using ND-1000 Nanodrop and Agilent 2200 TapeStation. the library for deep RNA sequencing was prepared according to a standard protocol established by RiboBio company in Guangzhou. The rRNAs were removed using Ribo-Zero™ Rrna Removal Reagent (Human/Mouse/Rat)-Illumina and library was prepared using NEBNext® Ultra™ Directional RNA Library Prep Kit for Illumina, USA NEB company. Then the paired-end 150 bp RNAs sequencing was performed using Illumina HiseqX-ten.

**Table 4:** The main clinical features of the asthmatic patients before and after desensitization treatment

No.	Gender	Age, years	Family history of allergies	Other sensibiligens	Before treatment				After treatment			
					Sampling time	Total IgE, IU/mL	DP	DF	Sampling time	Total IgE, IU/mL	DP	DF
E238–1	Female	15	/	/	2017.8.8	191	42.7	40.6	2017.12.9	323	65.2	71.6
E251–1	Male	15	F: AR	/	2017.8.25	1824	> 100	> 100	2018.1.31	1675	> 100	> 100
E241–1	Male	8	M: AR	/	2017.7.14	2604	> 100	/	2017.12.9	2099	> 100	> 100
E255–1	Male	13	F: AR; GM: DA (AMO)	/	2017.9.1	438	23.2	32.5	2018.1.31	421	27.9	31.8
E258-1B	Male	11	F: AR	Cockroach	2017.9.25	393	99.1	83.6	2018.4.2	283	52.2	61
E261–1	Male	9	/	/	2017.12.9	91.6	14.1	24.4	2018.5.8	129	11.8	24.7
E233–1	Male	12	/	/	2017.6.24	729	> 100	/	2017.10.21	723	85	79.8
E235–1	Male	16	F: AD+AR; M: AD	/	2017.6.10	323	47.7	51.3	2017.10.21	/	/	/
E257–1	Male	10	F: AR	/	2017.9.2	532	> 100	/	2017.12.23	761	> 100	91.2
E266–1	Male	10	M: AR + AD+FA	Cockroach, Crab, Shrimp	2018.3.17	936	96.4	/	2018.7.5	696	46.4	88.5

*F* Father;

*M* Mother;

*GM* Grandma;

*DA* Drug allergy;

*FA* Food allergy;

*DP* Dermatophagoides pteronyssinus;

*DF* Dermatophagoides farina;

### SCIT and drug treatments protocol

The patients were treated with conventional schedule injections of standardized aluminum formulated Der p extract (Alutard, ALK-Abelló, Hørsholm, Demark). The treatment protocol followed the recommended updosing schedule of 17 weeks before reaching a maintenance dose of 100,000 Alutard-SQ (Figure S6). As-needed use of short-acting bronchodilators (Salbutamol Sulfate Aerosol) for relieving asthma symptoms was also allowed.

### Basic bioinformatic analysis for identification of lncRNAs

The raw RNA-seq data were filtered using Trimmomatic v0.36 [50] (ILLUMINACLIP: TruSeq3-PE.fa:2:30:10:8:true SLIDINGWINDOW:4:15 LEADING:3 TRAILING:3 MINLEN:50) to remove low quality reads and adaptors. Then the clean reads were aligned to Ensembl hg38 human genome via STAR (v020201) [51]. Then with the help of program StringTie (v1.3.3b) [52], the output bam files were utilized to re-construct the new transcriptome for this study.

The assembled transcripts were subjected to stringent stepwise filtering pipeline to identify the high-confidence dataset of lncRNAs, which has been applied in our previous studies. Briefly, the known lncRNAs were initially extracted based on the “biotype_transcript” of reference gtf file of *Homo sapiens* from all the assembled transcripts via an in-house perl script. “biotype_transcript” tagged as “Long_non-coding_RNA”, “Non_coding”, “3prime_overlapping_ncRNA”, “Antisense”, “lincRNA”, “Retained_intron”, “Sense_intronic”, “Sense_overlapping”, “macro_lncRNA”, “bidirectional_promoter_lncRNA” was detected as lncRNAs.

For the remaining transcripts which were annotated by aligning against known protein sequences from NCBI nr database, Uniprot database, and the known human mRNAs derived from Ensembl database were excluded. This step aims at eliminating protein-coding sequences as much as possible. The remaining unmapped transcripts were further filtered to remove the sequences with length less than 200 nt and longest ORF longer than 100 residues. Then the second filtering round aimed at removing protein-coding sequences was conducted using Pfamscan [53] and CPC [54]. The remaining transcripts were retained as final dataset of lncRNAs.

### Differential expression analysis

All the assembled transcripts for each individual were quantified by featurecounts (v1.5.3) [55]. The expression profile was normalized using median of ratios method by R package DESeq2 [22]. Then the differential expression analysis was performed using pair-wise method of DESeq2 to detect the differentially expressed transcripts (DETs). Functional enrichment analysis for these DETs were conducted based on Metascape serve [56].

### Network analysis predicts lncRNA functions

The normalized expression profile was subjected to a R package WGCNA [57] for the weighted co-expression network analysis. Briefly, the function *softConnectivity* from WGCNA was used and the “randomly selected genes” parameter set at 5000, the power parameter pre-calculated by pickSoft-Threshold function of WGCNA. These analyses aim at detection of trait-related modules. Then the selected module with highly correlated RNAs were subjected to Metascape to search for enriched biological processes and pathways. The intramodular connectivity of mRNA and lncRNA in trait-related modules was assessed using Pearson Correlation Coefficient (PCC) method, |cor| > =0.7, *p*-value<=0.05.

### Validation by qRT-PCR

To validate the RNA expression level for the lncRNAs or mRNAs, we determined the expression levels of several selected lncRNA or mRNA transcripts by RT-qPCR in two different statuses (before and after treatment), including KLRK1, LINC02145 and GUSBP2. Primers were designed and synthesized by RiboBio company in Guangzhou. Upstream primer of KLRK1 is: 5′- CCGACACAAAGTCCCACACTC − 3′, downstream primer: 5′- AGATGCTTGCCTAAACGCCT − 3′. Upstream primer of LINC02145 is: 5′-AAGTACTGTTCTGCCTTCCACAC-3′, downstream primer: 5′-TGCCTTGGAAAGGTAGCGAG-3′. Upstream primer of GUSBP2 is: 5′- GAGCAGTACCATCTGGGTCTGG-3′, downstream primer: 5′- ACTTCATCTTGGATTTCCAGCCT-3′. Prepare 10 μl SYBR Green qRT-PCR kit (Soochow GenePharma Co. Ltd.) reaction system: cDNA (500 ng/20 μl): 1 μl, forward primer (10 pmol/L): 0.4 μl, reverse primer (10 pmol/L): 0.4 μl, 2 × SYBR Green PCR Master Mix 5 μl, RNase-free H_2_O 3.2 μl. Reaction condition: 95 °C 10 min heating, 95 °C 15 s denaturing, 60 °C 30 s annealing, 70 °C 30 s extension, 40 cycles. All reactions had 3 duplicates, CCAT1, E-cadherin and N-cadherin expression in colorectal tissue was calculated with 2^-ΔΔCt^ method. One-side T-test was used to test whether there is a difference in the expression level between groups before and after desensitization treatment. These analyses were achieved on the open source statistical programing language R. Concretely, the function shapiro.test() was used to test whether two groups before and after desensitization treatment are normal distribution. The function var.test() was used to test whether two groups are equal variance. The function t.test() was used to perform paired t-test for two groups.

### GEO data

The expression profile data for the validation of GUSBP2 and KLRK1 was downloaded from NCBI GEO database, which contains 45 alveolar macrophages from human subjects (30 non-smoker healthy people and 15 asthmatic patients).

## Supplementary information

**Additional file 1: Table S1.** Basic statistics of deep RNA sequencing data before and after processing.

**Additional file 2: Table S2.** Basic statistics of clean reads alignment against to the *Homo sapiens* genome sequence.

**Additional file 3: Table S3.** Basic statistics of assembly results of transcriptome in *Homo sapiens.*

**Additional file 4: Table S4.** Location of novel identified lncRNAs.

**Additional file 5: Figure S1.** Sample clustering to detect outliers. All the samples were in the clusters, all samples have passed the cuts.

**Additional file 6: Figure S2.** Analysis of network topology for various soft-thresholding powers. The left panel indicates the scale-free fit index (y-axis) as a function of the soft-thresholding power (x-axis). The right panel shows the mean connectivity (degree, y-axis) as a function of the soft-thresholding power (x-axis).

**Additional file 7: Figure S3.** Clustering dendrograms of transcripts, with dissimilarity based on topological overlap, together with assigned module colors.

**Additional file 8: Figure S4.** Visualizing the gene network using a heatmap plot. Light color represents low overlap and progressively darker red color represents higher overlap.

**Additional file 9: Figure S5.** Scatterplots of Gene Significance (GS) for recurrence vs Module Membership (MM) in the desensitization treatment-related module.

**Additional file 10: Figure S6.** The treatment protocol applied in the present study.

**Additional file 11: Figure S7.** QRT-PCR validation for three selected key lnRNAs or mRNAs. *P* value was calculated by paired T-test.

**Additional file 12: Figure S8.** Expression comparison between GUSBP2 and KLRK1 in GEO GSE2125. P value was calculated by unpaired T-test.

## Data Availability

All the raw data used in this study is deposited at DDBJ/EMBL/GenBank under the SRA accession PRJNA552157, associated with the BioSample SAMN12179172-SAMN12179191.

## Abbreviations

LncRNA: Long noncoding RNA
ASM: Airway smooth muscle
TH1: T helper cell 1
ORF: Open reading frame
AS: Alternative splicing
T_H_ cell: Helper T cell
MBD1: Methyl CpG binding protein 1
Aire: Autoimmune regulator
PCC: Pearson Correlation Coefficient
TLR3/4: Toll-like receptor3/4
CD: Crohn’s disease
PBMC: Peripheral blood mononuclear cell
DETs: Differentially expressed transcripts
GO: Gene Ontology; nr database
NCBI: Non-redundant database
