# Supplementary material for: Transcriptome analysis of peripheral whole blood identifies crucial lncRNAs implicated in childhood asthma
Source: BMC Med Genomics. 2020 Sep 18;13:136. doi: 10.1186/s12920-020-00785-y (PMC7501638; doi:10.1186/s12920-020-00785-y)
Supplement: Supplementary file 1 — Additional file 1: Table S1. Basic statistics of deep RNA sequencing data before and after processing. [file 12920_2020_785_MOESM1_ESM.docx]

**Table S1. Basic statistics of deep RNA sequencing data before and after processing.**

| **Sample ID** | **Status** | **Raw reads** | **Raw base** | **Clean reads** | **Clean base** |
| --- | --- | --- | --- | --- | --- |
| E233-1 | P_1, Treatment Before | 117,578,322 | 17,636,748,300 | 112,684,248 | 16,019,146,490 |
| E233-2 | P_1, Treatment After | 116,358,570 | 17,453,785,500 | 107,339,396 | 14,846,726,750 |
| E235-1 | P_2, Treatment Before | 135,178,314 | 20,276,747,100 | 128,646,286 | 18,289,889,168 |
| E235-2 | P_2, Treatment After | 120,410,784 | 18,061,617,600 | 112,713,942 | 15,755,242,130 |
| E238-1 | P_3, Treatment Before | 138,529,300 | 20,779,395,000 | 130,837,276 | 18,264,848,352 |
| E238-2 | P_3, Treatment After | 124,527,092 | 18,679,063,800 | 121,850,370 | 17,291,078,482 |
| E241-1 | P_4, Treatment Before | 136,480,008 | 20,472,001,200 | 133,454,182 | 18,946,396,236 |
| E241-2 | P_4, Treatment After | 114,874,846 | 17,231,226,900 | 111,948,602 | 15,889,539,106 |
| E251-1 | P_5, Treatment Before | 138,820,740 | 20,823,111,000 | 133,976,576 | 18,874,202,334 |
| E251-2 | P_5, Treatment After | 142,712,018 | 21,406,802,700 | 135,389,604 | 19,391,306,096 |
| E255-1 | P_6, Treatment Before | 136,264,082 | 20,439,612,300 | 133,416,858 | 19,024,389,074 |
| E255-2 | P_6, Treatment After | 138,129,632 | 20,719,444,800 | 133,713,730 | 19,205,695,324 |
| E257-1 | P_7, Treatment Before | 120,434,884 | 18,065,232,600 | 113,342,594 | 15,862,215,282 |
| E257-2 | P_7, Treatment After | 144,396,834 | 21,659,525,100 | 136,128,670 | 19,044,558,150 |
| E258-1B | P_8, Treatment Before | 124,192,486 | 18,628,872,900 | 120,723,768 | 17,780,446,876 |
| ZPY-12592 | P_8, Treatment After | 95,163,696 | 14,274,554,400 | 89,839,238 | 12,775,238,904 |
| E261-1 | P_9, Treatment Before | 125,472,872 | 18,820,930,800 | 119,297,044 | 16,482,675,274 |
| E261-2 | P_9, Treatment After | 124,655,634 | 18,698,345,100 | 118,450,658 | 16,737,154,912 |
| E266-1 | P_10, Treatment Before | 140,157,834 | 21,023,675,100 | 134,353,370 | 19,123,662,594 |
| E278-1 | P_10, Treatment After | 113,385,978 | 17,007,896,700 | 105,705,272 | 14,797,662,546 |
