# Supplementary material for: Transcriptome analysis of peripheral whole blood identifies crucial lncRNAs implicated in childhood asthma
Source: BMC Med Genomics. 2020 Sep 18;13:136. doi: 10.1186/s12920-020-00785-y (PMC7501638; doi:10.1186/s12920-020-00785-y)
Supplement: Supplementary file 2 — Additional file 2: Table S2. Basic statistics of clean reads alignment against to the Homo sapiens genome sequence. [file 12920_2020_785_MOESM2_ESM.docx]

**Table S2. Basic statistics of clean reads alignment against to the *Homo sapiens* genome sequence.**

| **Sample ID** | **Status** | **Mapped reads** | **Mapped rate (%)** |
| --- | --- | --- | --- |
| E233-1 | P_1, Treatment Before | 86,251,360 | 76.54 |
| E233-2 | P_1, Treatment After | 88,476,258 | 82.43 |
| E235-1 | P_2, Treatment Before | 98,314,330 | 76.42 |
| E235-2 | P_2, Treatment After | 84,490,730 | 74.96 |
| E238-1 | P_3, Treatment Before | 115,994,484 | 88.66 |
| E238-2 | P_3, Treatment After | 97,983,746 | 80.41 |
| E241-1 | P_4, Treatment Before | 108,914,626 | 81.61 |
| E241-2 | P_4, Treatment After | 89,819,962 | 80.23 |
| E251-1 | P_5, Treatment Before | 111,202,310 | 83.00 |
| E251-2 | P_5, Treatment After | 111,447,220 | 82.32 |
| E255-1 | P_6, Treatment Before | 106,766,724 | 80.02 |
| E255-2 | P_6, Treatment After | 112,214,330 | 83.92 |
| E257-1 | P_7, Treatment Before | 87,159,846 | 76.90 |
| E257-2 | P_7, Treatment After | 109,678,534 | 80.57 |
| E258-1B | P_8, Treatment Before | 102,192,174 | 84.65 |
| ZPY-12592 | P_8, Treatment After | 62,518,864 | 69.59 |
| E261-1 | P_9, Treatment Before | 92,031,208 | 77.14 |
| E261-2 | P_9, Treatment After | 90,628,062 | 76.51 |
| E266-1 | P_10, Treatment Before | 104,879,994 | 78.06 |
| E278-1 | P_10, Treatment After | 87,875,034 | 83.13 |
