# Supplementary material for: Transcriptome analysis of peripheral whole blood identifies crucial lncRNAs implicated in childhood asthma
Source: BMC Med Genomics. 2020 Sep 18;13:136. doi: 10.1186/s12920-020-00785-y (PMC7501638; doi:10.1186/s12920-020-00785-y)
Supplement: Supplementary file 3 — Additional file 3: Table S3. Basic statistics of assembly results of transcriptome in Homo sapiens. [file 12920_2020_785_MOESM3_ESM.docx]

**Table S3. Basic statistics of assembly results of transcriptome in *Homo sapiens.***

| **Statistics terms** | **Number** | |
| --- | --- | --- |
| Total number | 215,331 |  |
| Total length of (bp) | 402,640,670 | |
| Average length (bp) | 1,869.87 | |
| N50 Length (bp) | 3,619 | |
| Maximum length (bp) | 341,588 | |
| Minimum length (bp) | 8 | |
| GC content (%) | 47.63 | |
