# Supplementary figures and images for: Transcriptome analysis of peripheral whole blood identifies crucial lncRNAs implicated in childhood asthma
Source: BMC Med Genomics. 2020 Sep 18;13:136. doi: 10.1186/s12920-020-00785-y (PMC7501638; doi:10.1186/s12920-020-00785-y)

# Sample clustering to detect outliers

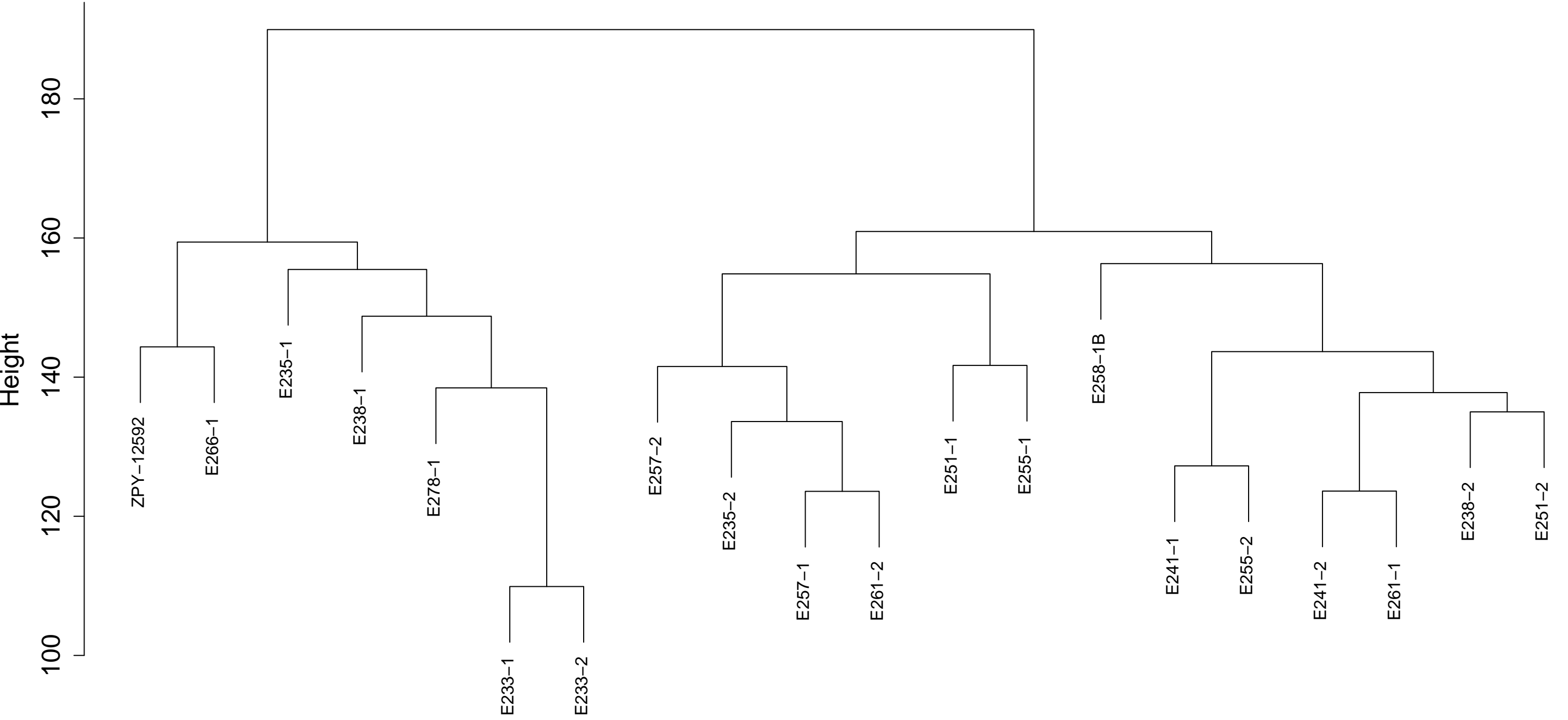

Supplement: Supplementary file 5 — Additional file 5: Figure S1. Sample clustering to detect outliers. All the samples were in the clusters, all samples have passed the cuts. [file 12920_2020_785_MOESM5_ESM.pdf]

### Scale independence

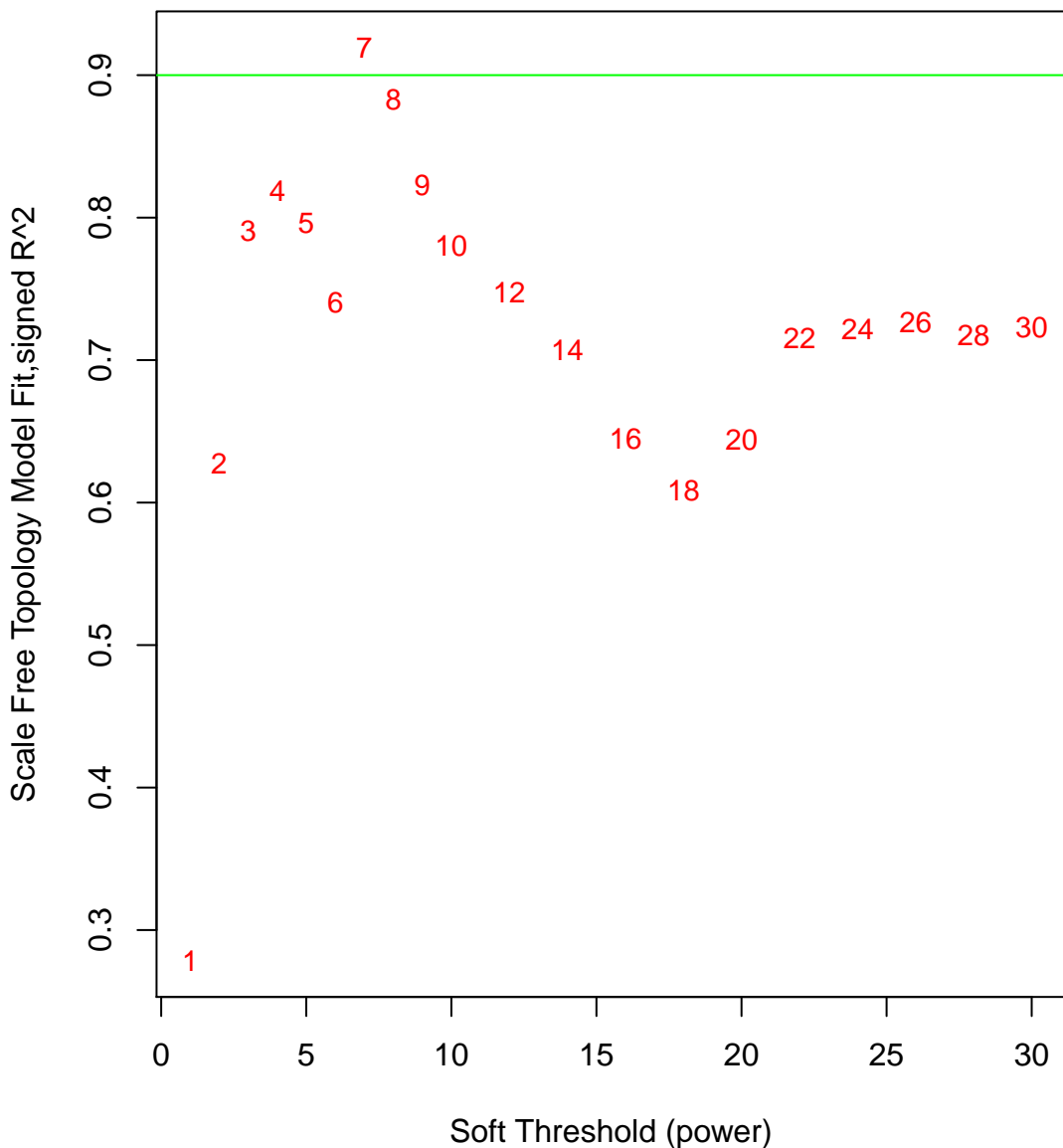

### Mean connectivity

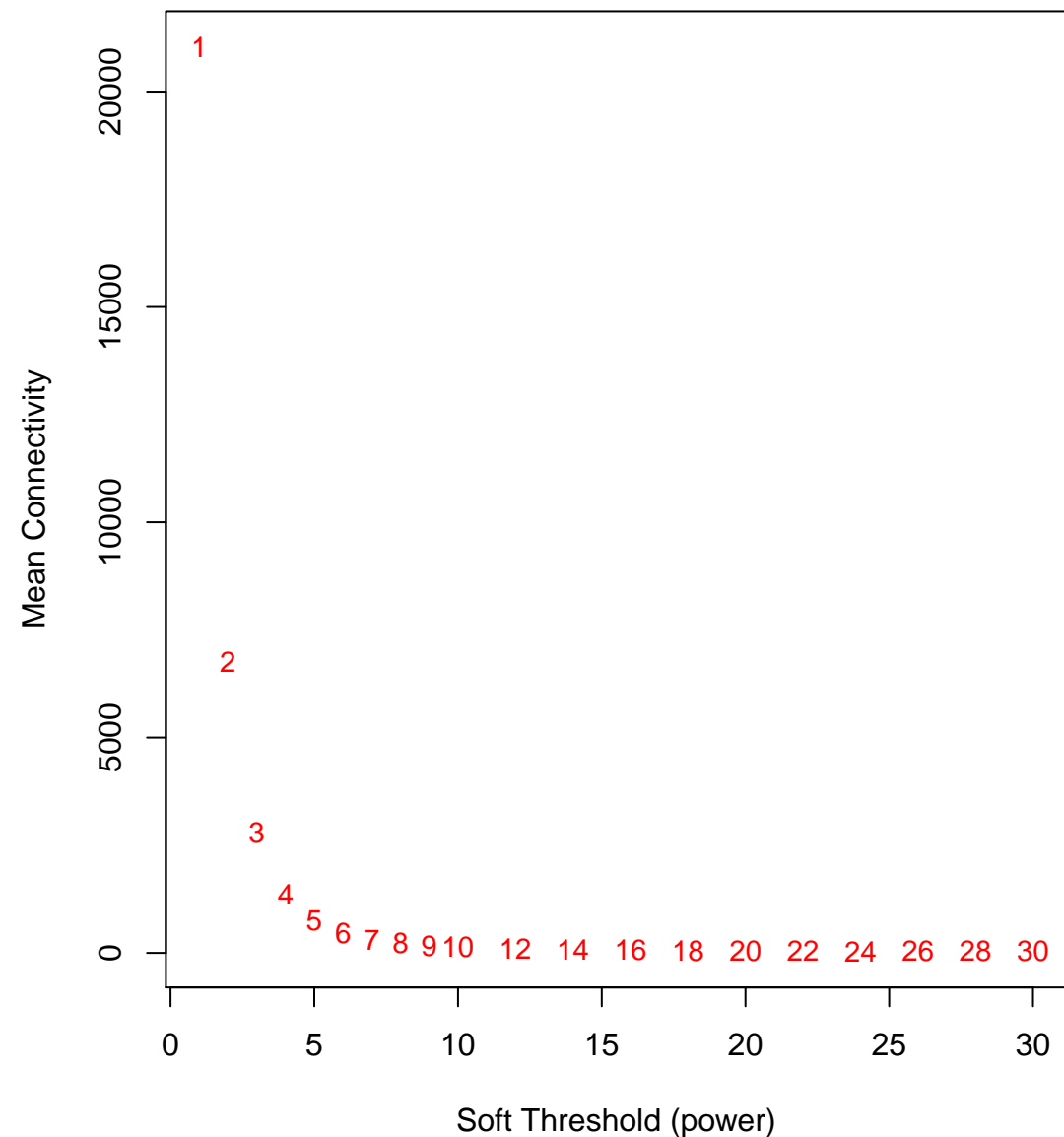

Supplement: Supplementary file 6 — Additional file 6: Figure S2. Analysis of network topology for various soft-thresholding powers. The left panel indicates the scale-free fit index (y-axis) as a function of the soft-thresholding power (x-axis). The right panel shows the mean connectivity (degree, y-axis) as a function of the soft-thresholding power (x-axis). [file 12920_2020_785_MOESM6_ESM.pdf]

Cluster Dendrogram

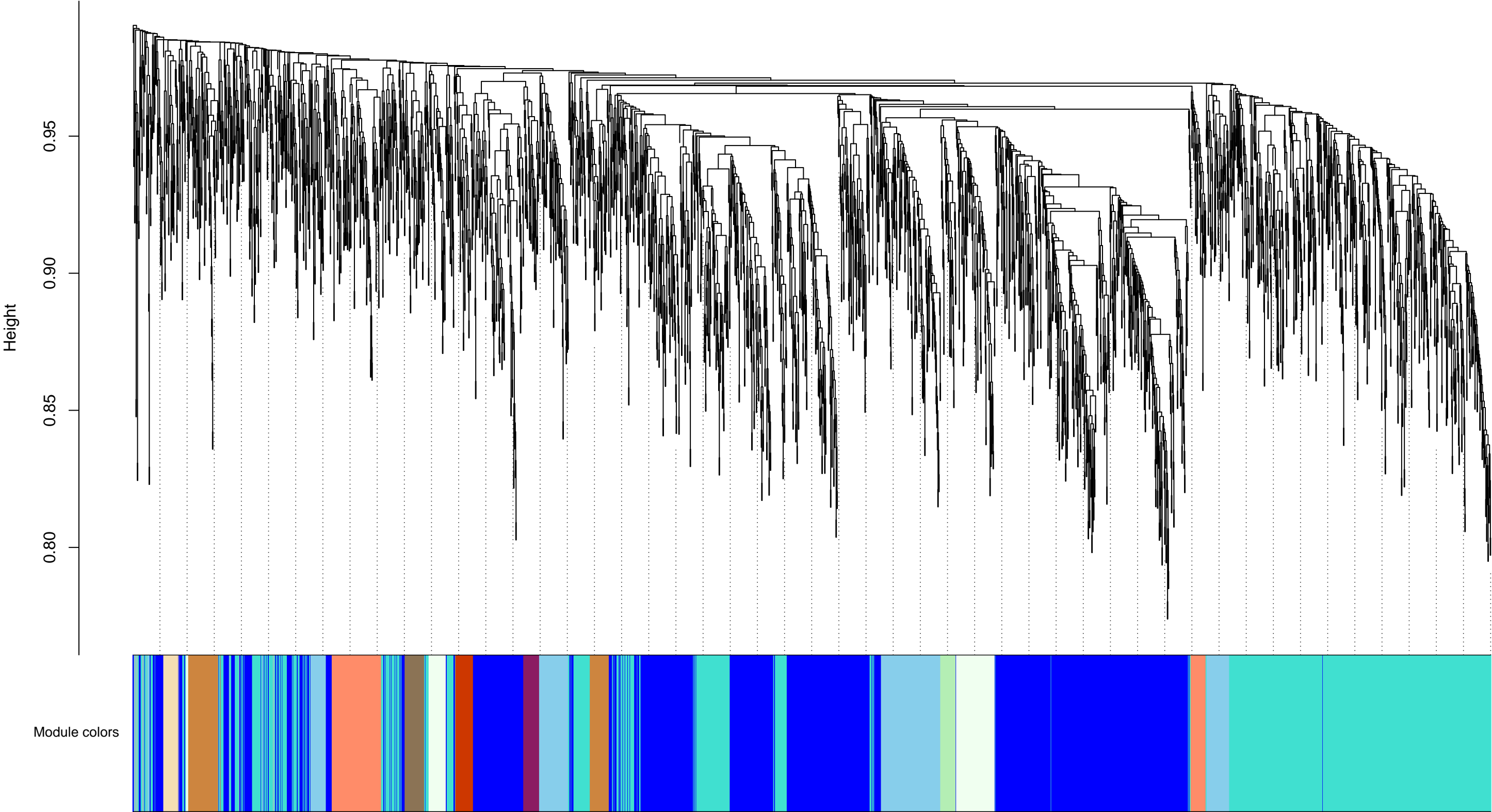

Supplement: Supplementary file 7 — Additional file 7: Figure S3. Clustering dendrograms of transcripts, with dissimilarity based on topological overlap, together with assigned module colors. [file 12920_2020_785_MOESM7_ESM.pdf]

Network heatmap plot, selected transcripts

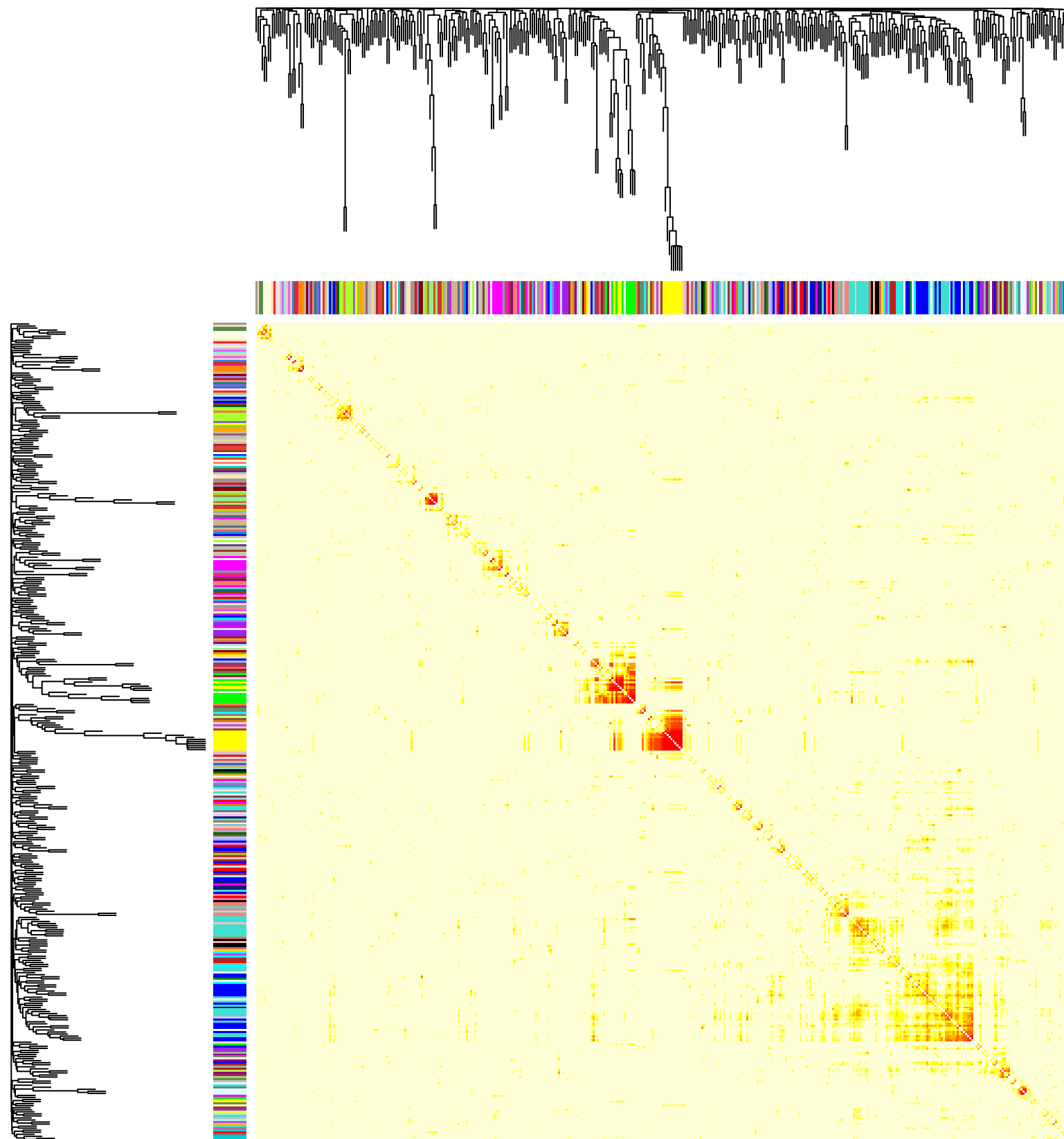

Supplement: Supplementary file 8 — Additional file 8: Figure S4. Visualizing the gene network using a heatmap plot. Light color represents low overlap and progressively darker red color represents higher overlap. [file 12920_2020_785_MOESM8_ESM.pdf]

Module membership vs. gene significance  
cor=0.39, p=0.0061

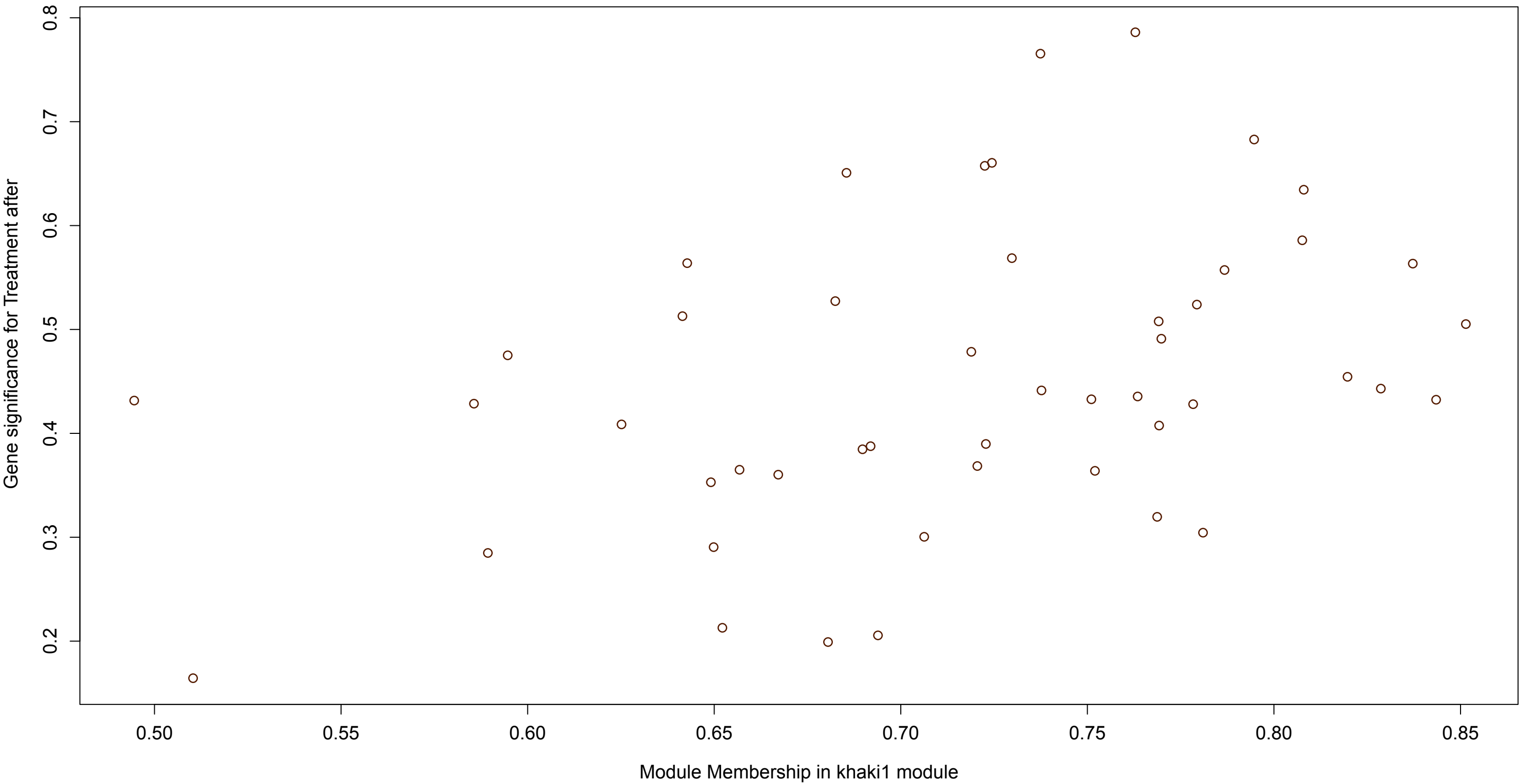

Supplement: Supplementary file 9 — Additional file 9: Figure S5. Scatterplots of Gene Significance (GS) for recurrence vs Module Membership (MM) in the desensitization treatment-related module. [file 12920_2020_785_MOESM9_ESM.pdf]

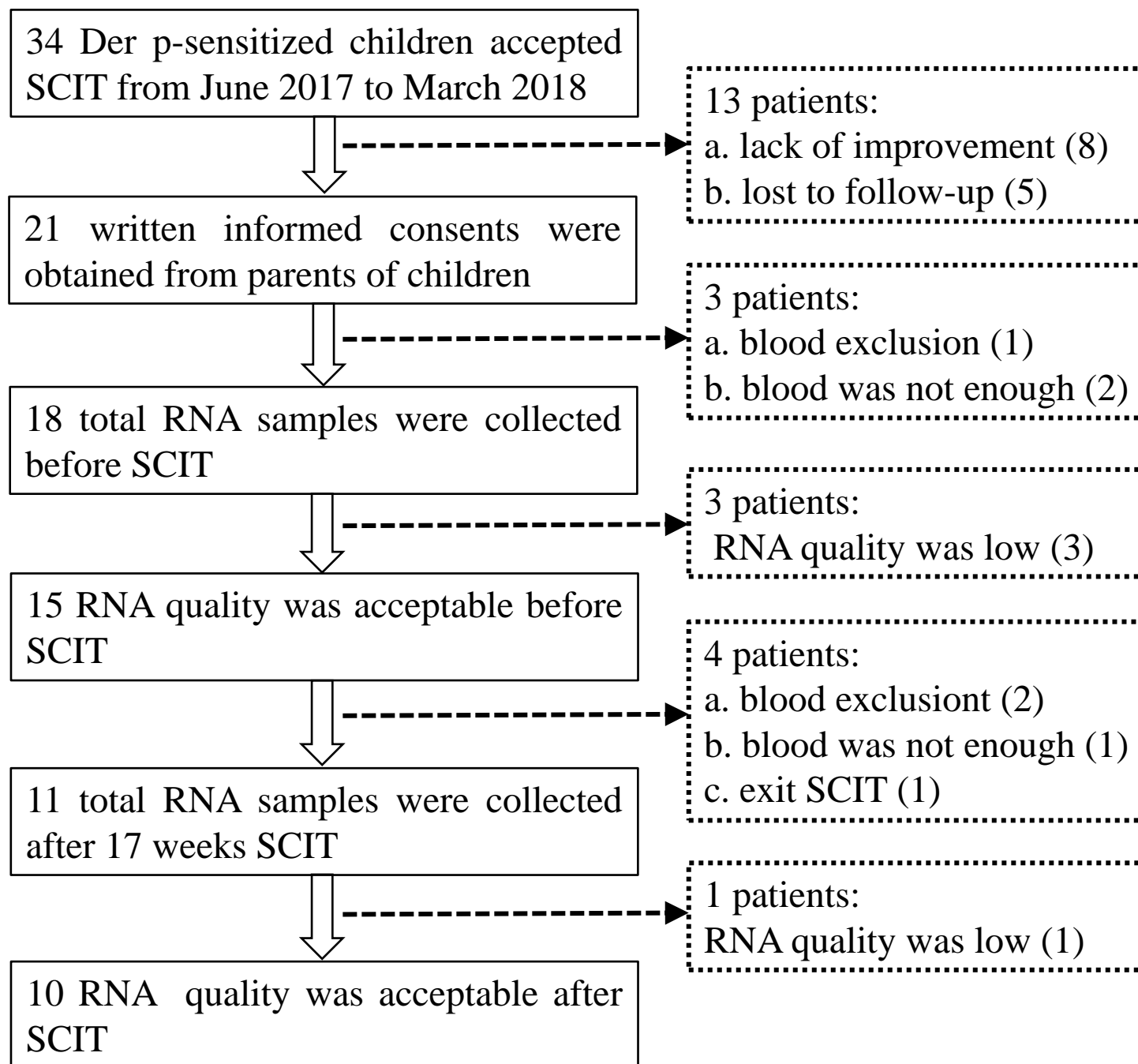

Supplement: Supplementary file 10 — Additional file 10: Figure S6. The treatment protocol applied in the present study. [file 12920_2020_785_MOESM10_ESM.pdf]

**A****GUSBP2**

before treatment  
after treatment

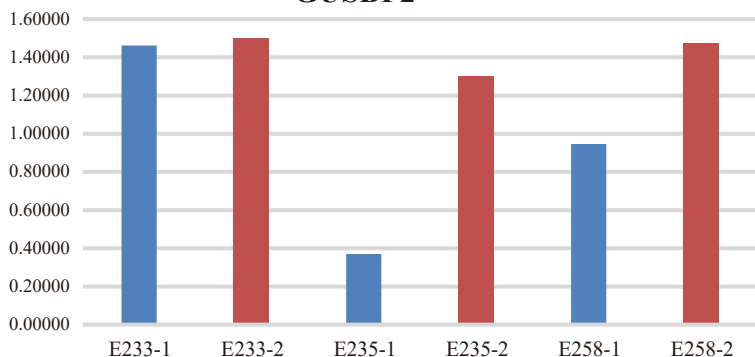**B****KLRK1**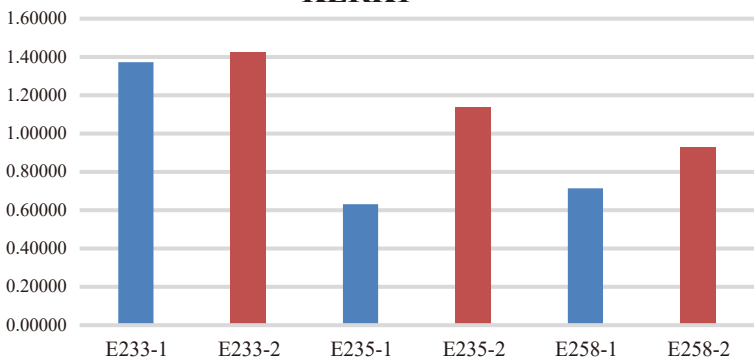**C****LINC02145**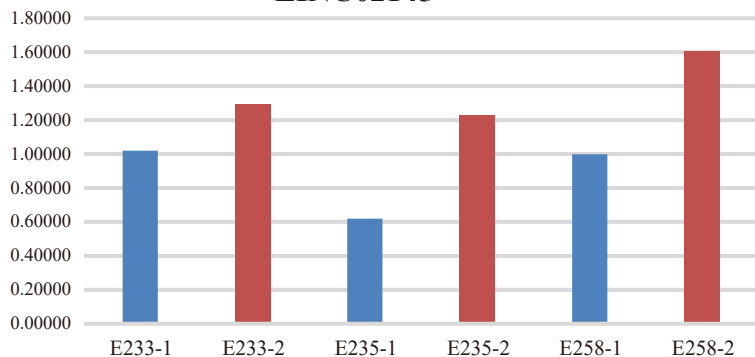

Supplement: Supplementary file 11 — Additional file 11: Figure S7. QRT-PCR validation for three selected key lnRNAs or mRNAs. P value was calculated by paired T-test. [file 12920_2020_785_MOESM11_ESM.pdf]

**A****GUSBP2**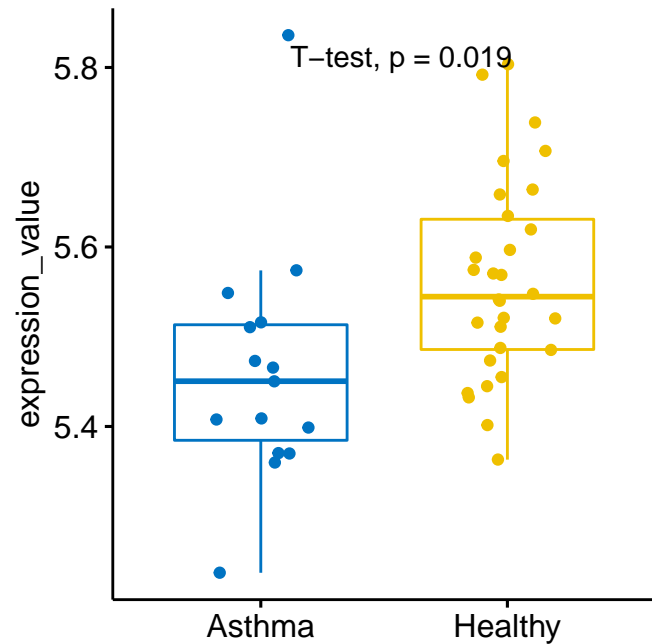**B****KLRK1**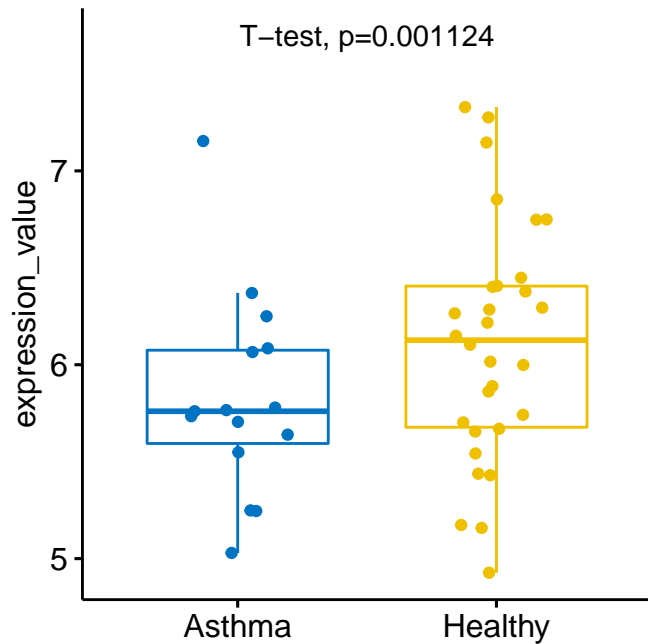

Supplement: Supplementary file 12 — Additional file 12: Figure S8. Expression comparison between GUSBP2 and KLRK1 in GEO GSE2125. P value was calculated by unpaired T-test. [file 12920_2020_785_MOESM12_ESM.pdf]
